# Latent Tracheobronchial Adenopathy

**Published:** 1914-08

**Authors:** 


**Latent Tracheobronchial Adenopathy.** Spéder & Dubourg recapitulate their paper in Archives D'Électricité Medicalé, 1914, No. 382, as follows:

I. Radiographic examination should be done from in front extra rapidly, best 1-100 second exposure, with a hard tube from the back.

2. All cases which showed clinical signs gave positive radiographic findings and radiography confirmed the diagnosis in cases in which the clinical signs were doubtful.

3. Radiography proved more exact than physical examination. Of the physical methods the best are the sign of d'Espine

(auscultation over the dorsal vertebrae) and Martin du Magny's sign (auscultation over the base).

4. Adenopathy is most frequently bilateral and in many cases intrapulmonary gangli will also be found.

5. In all cases which showed prevertebral ganglia by d'Espine's sign radiographic examination showed ganglia at the hilus as well.

6. Oblique examination is not of much value.

C. G. L-W.

